# Ethnic inequality in place of death: Analysis using ‘gold standard’ self-reported ethnicity data from the Census Longitudinal Study

**DOI:** 10.1177/02692163251395455

**Published:** 2025-12-04

**Authors:** J. M. Davies, K. C. Chua, M. Maddocks, F. E. M. Murtagh, K. E. Sleeman

**Affiliations:** 1 Cicely Saunders Institute, Department of Palliative Care, Policy and Rehabilitation, Faculty of Nursing, Midwifery and Palliative Care, King’s College London, UK; 2 Biostatistics and Health Informatics, Institute of Psychiatry, Psychology and Neuroscience, King’s College London, UK; 3 Wolfson Palliative Care Research Centre, Hull York Medical School, University of Hull, UK

**Keywords:** ethnicity, place of death, inequality, census

## Background

In high-income countries, people from ethnic minority backgrounds are less likely to access specialist palliative care^1^ and appropriate pain relief medication,^2^ and use unplanned hospital care more in the last months of life.^3^ Where people die depends on many social and clinical factors.^4^ Most people prefer to avoid dying in hospital if possible, and yet hospital remains the most common place of death.^4^

Historically, it has been challenging to explore ethnic inequality in place of death because ethnicity is not recorded on death certificates in most countries.^5^ Census data contains self-reported ethnicity data considered the ‘gold-standard’ compared to the assigned ethnicity information common in clinical records. When linked to mortality records, Census data provides a valuable but underused resource for understanding ethnic inequalities in place of death.^6^

We investigated the association between ethnicity and death in hospital (compared to all other locations) and examined how far this association is explained by geography, socioeconomic factors, and cause of death.

## Methods

Census data linked to mortality records for a 1% sample of the population in England and Wales who died between 1st January 2011 and 31st December 2017, accessed through the Office for National Statistics Longitudinal Study (ONS LS). Death in hospital (vs home, hospice, and care home) was the outcome. Ethnicity was the main exposure coded in 11 categories. We ran Poisson models with robust standard errors, separately for men and women, adjusted for age, and cumulatively adjusted for other explanatory factors.

Analysis was carried out in Stata SE v17.0, with code available here: https://github.com/joannamariedavies/ONS_LS.

## Results

After excluding 688 people who died in ‘other’ locations, and 17 with missing place of death, 34,230 decedents were included. Table 1 describes the sample.

**Table 1.** Sample characteristics for decedents from the ONS Longitudinal Study (deaths between 2011 and 2017), by place of death.

Numbers are *n* (%) unless otherwise stated	Overall (column %)	Hospital (row %)	Home, hospice, care home (row %)
*N*	34,230	16,929 (49.5)	17,301 (50.5)
Age at death, median (IQR)	82 (72, 89)	81 (72, 88)	82 (72, 89)
Gender			
Male	16,338 (47.7)	8422 (51.5)	7916 (48.5)
Female	17,892 (52.3)	8507 (47.5)	9385 (52.5)
Ethnicity			
White British	31,281 (91.4)	15,245 (48.7)	16,036 (51.3)
Irish	600 (1.8)	302 (50.3)	298 (49.7)
White Other	633 (1.8)	333 (52.6)	300 (47.4)
Mixed	138 (0.4)	67 (48.6)	71 (51.4)
Indian	548 (1.6)	365 (66.6)	183 (33.4)
Pakistani	264 (0.8)	167 (63.3)	97 (36.7)
Bangladeshi	90 (0.3)	69 (76.7)	21 (23.3)
Black African	91 (0.3)	58 (63.7)	33 (36.3)
Black Caribbean	279 (0.8)	137 (49.1)	142 (50.9)
Chinese	7* (*.*)	3* (*.*)	3* (*.*)
Other	218 (0.6)	142 (65.1)	76 (34.9)
Missing	* (*.*)	* (*.*)	* (*.*)
Region			
North East	1784 (5.2)	901 (50.5)	883 (49.5)
North West	4612 (13.5)	2383 (51.7)	2229 (48.3)
Yorkshire and The Humber	3475 (10.2)	1666 (47.9)	1809 (52.1)
East Midlands	2841 (8.3)	1401 (49.3)	1440 (50.7)
West Midlands	3445 (10.1)	1778 (51.6)	1667 (48.4)
East of England	3678 (10.7)	1703 (46.3)	1975 (53.7)
London	3428 (10.0)	1899 (55.4)	1529 (44.6)
South East	5238 (15.3)	2390 (45.6)	2848 (54.4)
South West	3609 (10.5)	1580 (43.8)	2029 (56.2)
Wales	2120 (6.2)	1228 (57.9)	892 (42.1)
Rural/urban			
1. Most urban	15,707 (45.9)	8099 (51.6)	7608 (48.4)
2. Very urban	2953 (8.6)	1438 (48.7)	1515 (51.3)
3. Urban	5290 (15.5)	2587 (48.9)	2703 (51.1)
4. Rural	7481 (21.9)	3569 (47.7)	3912 (52.3)
5. Most rural	2799 (8.2)	1236 (44.2)	1563 (55.8)
Area-based deprivation (Index of Multiple Deprivation, 2015)			
1 (most deprived)	6944 (20.3)	3664 (52.8)	3280 (47.2)
2	6721 (19.6)	3524 (52.4)	3197 (47.6)
3	7245 (21.2)	3461 (47.8)	3784 (52.2)
4	6905 (20.2)	3275 (47.4)	3630 (52.6)
5	64** (<19)	30** (*.*)	34** (*.*)
Missing	(<1)^a^	(*.*)^a^	(*.*)^a^
Occupation-based social class^b^			
Employers in large establishments/higher managerial	617 (1.8)	289 (46.8)	328 (53.2)
Higher professionals	1594 (4.7)	765 (48)	829 (52)
Lower professionals and technical	5353 (15.6)	2556 (47.7)	2797 (52.3)
Intermediate	4485 (13.1)	2224 (49.6)	2261 (50.4)
Employers in small organisations	3205 (9.4)	1552 (48.4)	1653 (51.6)
Lower supervisory	3286 (9.6)	1689 (51.4)	1597 (48.6)
Semi routine	5861 (17.1)	2959 (50.5)	2902 (49.5)
Routine	6323 (18.5)	3349 (53)	2974 (47)
Never worked/long-term unemployed	3396 (9.9)	1500 (44.2)	1896 (55.8)
Missing	110 (0.3)	46 (41.8)	64 (58.2)
Highest level of educational qualification			
No academic or professional qualifications	20,119 (58.8)	10,026 (49.8)	10,093 (50.2)
Level 1: 1–4 O Levels/CSE/GCSEs, Entry Level	1867 (5.5)	956 (51.2)	911 (48.8)
Level 2: 5+ O Level/CSEs/GCSEs, School Certificate	2362 (6.9)	1174 (49.7)	1188 (50.3)
Apprenticeship	1679 (4.9)	848 (50.5)	831 (49.5)
Level 3: 2+ A Levels/VCEs, Higher School Certificate	12** (*.*)	62* (*.*)	65* (*.*)
Level 4+: Degree (BA, BSc), Higher Degree	4815 (14.1)	2248 (46.7)	2567 (53.3)
Other: Vocational or foreign qualifications	2093 (6.1)	1046 (50)	1047 (50)
Missing	(*.*)^a^	(*.*)^a^	(*.*)^a^
English or Welsh language proficiency			
Main language is English or Welsh	32,909 (96.1)	16,102 (48.9)	16,807 (51.1)
Speaks very well	260 (0.8)	158 (60.8)	102 (39.2)
Speaks well	393 (1.1)	241 (61.3)	152 (38.7)
Speaks not very well	439 (1.3)	283 (64.5)	156 (35.5)
Does not speak English or Welsh	21* (*.*)	13* (*.*)	7* (*.*)
Missing	(*.*)^a^	(*.*)^a^	(*.*)^a^
Underlying cause of death			
Cancer	9880 (28.9)	3802 (38.5)	6078 (61.5)
Cardiovascular	8552 (25.0)	4670 (54.6)	3882 (45.4)
Dementia	3691 (10.8)	1006 (27.3)	2685 (72.7)
Respiratory	2028 (5.9)	1304 (64.3)	724 (35.7)
Other	1759 (5.1)	719 (40.9)	1040 (59.1)
Sudden causes	8320 (24.3)	5428 (65.2)	2892 (34.8)
Place of death			
Hospital	16,929 (49.5)		
Home	8005 (23.4)	-	-
Care home	7345 (21.5)	-	-
Hospice	1951 (5.7)	-	-

Data source: ONS Longitudinal Study.

a Cell counts of <10 have been supressed, where * appears after a digit (e.g. 7*) this number has been supressed to mask small cell counts in another row.

b Derived from a question about ‘your main job or, if not working, your last main job’ using the National Statistics Socio-Economic Classifications (NS-SEC), a standard UK system that classifies individuals into socio-economic groups based on their occupation, employment status, and supervisory responsibilities. Example occupations include - employers in large establishments/higher managerial (directors of major organisations, senior officers in government); higher professionals (medical practitioners, solicitors); lower professional (teachers, nurses); intermediate (medical secretaries, police officers); employers in small organisations (farmers, window cleaners); semi-routine (dental nurses, traffic wardens); routine (hairdressers, waiting staff).

**Figure 1.**
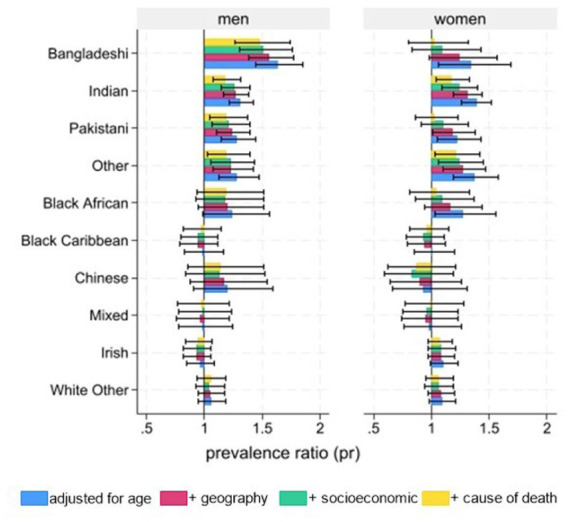
Adjusted prevalence ratio and 95% confidence intervals for death in hospital (compared to home, hospice or care home), by ethnic group and sex, compared to white British. Data source: ONS Longitudinal Study. *Note.* 1. Poisson models with robust standard errors adjusting for: **model 1 (age)**: age (numerical); **model 2 (geography)**: geographical region (categorical), level of urbanicity (numerical); **model 3 (socioeconomic)**: deciles of area-based deprivation (numerical), occupation-based social class (numerical), highest educational qualification (categorical), English/Welsh language proficiency (numerical); **model 4 (cause of death)**: underlying cause of death (categorical). 2. ‘Other’ ethnic groups include Asian Other, Black Other, Arab, and other ethnic group categories. 3. For men: model 1 & 2 *n* = 16,333; model 3 & 4 *n* = 16,285. For women: model 1 & 2 *n* = 17,882; model 3 & 4 *n* = 17,830.

After adjusting for age, geography, socioeconomic factors, and underlying cause of death, Bangladeshi men (PR 1.48; 95% CI 1.26-1.74), Pakistani men (1.19; 1.04–1.37), Indian men (1.18; 1.07–1.31) and women (1.17; 1.04–1.33), and men (1.19; 1.02–1.39) and women (1.21; 1.03–1.42) from ‘Other’ ethnic groups have statistically significantly higher rates of death in hospital, compared to White British men and women (Figure 1).

## Discussion

In this representative sample of people who died in England and Wales, most ethnic minority groups have a higher proportion of deaths in hospital than White British people. For some groups, these differences are explained by differences in age and other factors. However, for Bangladeshi and Pakistani men, Indian men and women, and men and women from ‘Other’ ethnic groups, higher rates of death in hospital remain statistically significant after adjusting for differences in age, geography, socioeconomic factors, and underlying cause of death.

A strength of our analysis is that we used self-reported ethnicity data from the Census. Data linkage across primary and secondary health records can improve the quality of ethnicity information in routinely collected patient records.^7^ However, there remain persistent inaccuracies in the ethnicity information recorded in health records and these inaccuracies are more common for minority groups.^8^ For historical reasons associated with the Holocaust, in some countries, including France and Germany, the collection of ethnicity data is highly restricted, leading to ‘ethnic invisibility’ in administrative and survey data. In England and Wales, ethnicity has recently been introduced to death certificates, assigned by the attending practitioner based on the patient record. The usability of this data will depend on the quality and completeness of the patient record.^8,9^

Our sample size allowed us to use refined categories of ethnicity and sex, revealing important differences between subgroups, and we included good quality sociodemographic and geographical data. Potentially important unmeasured confounders include symptom burden, preferences, and access to community services. A limitation is that our data is relatively old (deaths between 2011 and 2017) but at the time of writing, the 2021 census was not available in the ONS Longitudinal Study. We used an area-based measure of socio-economic deprivation, which may not reflect individuals’ experience. Despite a large overall sample size, small numbers in some of the ethnic minority groups prevented further intersectional analysis. Analysis of census data linked to mortality data for the full population would offer more opportunities to explore interactions between ethnicity and deprivation, and geographical variation in ethnic inequalities in place of death.

This analysis highlights an ethnic inequality in place of death and differences between genders not previously described. Our findings are nuanced, illustrating how some ethnic differences in place of death are explained by differences in age and other factors, while others are not. Our findings align with recent analyses highlighting South Asian communities as being at higher risk of unplanned hospital-based care in the last months of life.^3^ There is a lack of research on the reasons for these inequalities and whether they represent inequity in access to services.^10^ Important first steps are to measure and monitor ethnic inequalities in palliative and end-of-life care, and to improve the quality of ethnicity information in routinely collected health records.^10^ This should include public discussion on the purpose of collecting data on race and ethnicity. In addition, more work is needed to understand the experiences, needs and preferences of people from ethnic minority communities living with terminal illness to inform more equitable care.
